# Endothelin receptor antagonist and airway dysfunction in pulmonary arterial hypertension

**DOI:** 10.1186/1465-9921-10-129

**Published:** 2009-12-30

**Authors:** Annette S Droste, David Rohde, Mirko Voelkers, Arthur Filusch, Thomas Bruckner, Mathias M Borst, Hugo A Katus, F Joachim Meyer

**Affiliations:** 1 Department of Cardiology, Angiology and Respiratory Medicine, Heidelberg, Germany; 2 Institute for Medical Biometry and Informatics of the University, Heidelberg, Germany

## Abstract

**Background:**

In idiopathic pulmonary arterial hypertension (IPAH), peripheral airway obstruction is frequent. This is partially attributed to the mediator dysbalance, particularly an excess of endothelin-1 (ET-1), to increased pulmonary vascular and airway tonus and to local inflammation. Bosentan (ET-1 receptor antagonist) improves pulmonary hemodynamics, exercise limitation, and disease severity in IPAH. We hypothesized that bosentan might affect airway obstruction.

**Methods:**

In 32 IPAH-patients (19 female, WHO functional class II (n = 10), III (n = 22); (data presented as mean ± standard deviation) pulmonary vascular resistance (11 ± 5 Wood units), lung function, 6 minute walk test (6-MWT; 364 ± 363.7 (range 179.0-627.0) m), systolic pulmonary artery pressure, sPAP, 79 ± 19 mmHg), and NT-proBNP serum levels (1427 ± 2162.7 (range 59.3-10342.0) ng/L) were measured at baseline, after 3 and 12 months of oral bosentan (125 mg twice per day).

**Results and Discussion:**

At baseline, maximal expiratory flow at 50 and 25% vital capacity were reduced to 65 ± 25 and 45 ± 24% predicted. Total lung capacity was 95.6 ± 12.5% predicted and residual volume was 109 ± 21.4% predicted. During 3 and 12 months of treatment, 6-MWT increased by 32 ± 19 and 53 ± 69 m, respectively; p < 0.01; whereas sPAP decreased by 7 ± 14 and 10 ± 19 mmHg, respectively; p < 0.05. NT-proBNP serum levels tended to be reduced by 123 ± 327 and by 529 ± 1942 ng/L; p = 0.11). There was no difference in expiratory flows or lung volumes during 3 and 12 months.

**Conclusion:**

This study gives first evidence in IPAH, that during long-term bosentan, improvement of hemodynamics, functional parameters or serum biomarker occur independently from persisting peripheral airway obstruction.

## Introduction

Idiopathic pulmonary arterial hypertension (IPAH) is a rare pulmonary vasculopathy of unknown origin [1]. Patients with IPAH are often severely compromised by dyspnea, exercise intolerance and progressive right ventricular failure [1].

In 171 IPAH patients, significant peripheral airway obstruction independently from pulmonary hemodynamics has been described [2]. Given the proximity of pulmonary vasculature and peripheral airways, coupling between the pulmonary blood vessels and airways has partially been attributed to mechanical forces due to shared structural changes or vascular rigidity [3]. Moreover in IPAH, the imbalance favoring mediators of increased vascular smooth muscle tone and proliferation in the affected vessels adjacent to small airways are suggested underlying pathomechanisms [4]. Endothelin-1 (ET-1) is a potent mediator of both vaso- and bronchoconstriction [5]. ET-1 overexpression was found in lung tissue [6] and in plasma of IPAH patients in correlation with disease severity and prognosis [7].

Thus, supported by evidence of the pathogenic role of ET-1, the ET-1 receptor blockade has become a prominent and established approach to treat IPAH patients. Bosentan is a dual ET-1 receptor antagonist approved for the treatment of IPAH patients of functional class III (Europe) and II-IV (USA and Canada), and is now available in many parts of the world [8]. Bosentan has been shown to improve pulmonary hemodynamics, right heart function, exercise tolerance, and time to clinical worsening [9,10].

Moreover, in animal studies, bosentan prevented an ET-1-induced decrease in airway conductance and the blunted bronchial responsiveness to metacholine [11]. It is, however, unclear whether long-term ET-1 receptor blockade influences peripheral airways obstruction in patients with IPAH.

Therefore, this study was designed to investigate peripheral airway function in correlation to severity of IPAH during long-term treatment with bosentan. In 32 consecutive IPAH patients, lung mechanics, pulmonary haemodynamics, six-minute walk distance, and biomarkers were assessed before, during 3 and 12 months of therapy.

## Materials and methods

### Study population and medication

This study was conducted in a university tertiary referral center for patients with pulmonary hypertension (Dept of Cardiology and Respiratory Medicine, Medical Center, University Hospital, Heidelberg, Germany) and included patients with IPAH [1]. The diagnosis of IPAH was made after right heart catheterization at rest, and ventilation-perfusion scan, spiral computer tomography, three-dimensional angiography magnetic resonance tomography, or pulmonary angiography to rule out pulmonary embolic etiology, and after exclusion of underlying autoimmune disease, collagen vascular disease, hepatic or HIV infection, and nocturnal deoxygenation.

None of the patients was on bronchodilator treatment or had a history or signs of lung disease. Patients receiving beta-blockers were not included. Patients with clinical or radiological signs of cardiopulmonary decompensation were not included. None of the patients was active smoker and 7 had smoked in the past.

On inclusion, all patients were without specific pulmonary vasoactive therapy, including endothelin receptor blockade, phosphodiesterase inhibition, or prostanoids. After baseline measurements, treatment with bosentan was initiated as recommended: i.e. oral bosentan 62.5 mg twice daily, and after 4 weeks target dose of bosentan was 125 mg twice daily for the remaining study period including therapy monitoring as recommended.

The study was approved by the local ethics committee (Votum 301/2008), and written informed consent from the patients was weaved by the local ethics committee. The study was in accordance with the recommendations found in the Helsinki Declaration.

### Echocardiography

Transthoracic echocardiography was performed in the left decubitus or supine position using commercially available ultrasound equipment (Phillips iE 33, Philips Ultrasound, Bothell, Washington, USA). Systolic PAP was measured as described previously [12].

### Pulmonary function

Spirometry and body plethysmography (Cardinal Health, Viasys, Erich Jaeger, MasterLabPro, Wuerzburg, Germany) were performed according to standard protocols [13]. Lung function reference values corrected for sex, age, and height were used [13,14].

### Serum biomarker

Blood samples were drawn from a peripheral vein and analyzed for N-terminal-pro-B-type natriuretic peptide (NT-proBNP) serum levels using a commercially available assay (Roche Elecsys proBNP; Roche Diagnostics; Mannheim, Germany).

### Six-minute walk test

The results of the six-minute walk test (6-MWT) were counted from the laps achieved on a 60-m course in a straight hospital hallway that was seldom used. The test equipment and the interaction with the patient were provided as recommended [15].

### Data analysis

Statistical analysis was performed by a professional statistician using standard software (SAS 9.1 WIN). Results are expressed as mean ± standard deviation (SD). Paired and unpaired Student's t-test and Pearson's correlation coefficient were analysed as appropriate. P-values < 0.05 were considered statistically significant.

## Results

### Exercise tolerance and pulmonary hemodynamics

The 32 consecutive patients (19 female, 59% of patients) with the age of 56.4 ± 14.7 (21-81) had moderate to severe IPAH.

During right heart catheterization at rest, mean pulmonary artery pressure (PAP) was elevated to 49 ± 17 mmHg (range 27 to 85 mmHg), pulmonary vascular resistance (PVR) was increased to 10.8 ± 5.1 Wood units (range 4 to 21 Wood units), and cardiac output (CO) was decreased to 3.8 ± 1.3 L × min^-1 ^(range 2.2 to 7.2 L × min^-1^).

In accordance, systolic pulmonary arterial pressure (sPAP) assessed during echocardiography was elevated to more than twice the normal limit (79 ± 19 mmHg). After 3 months of treatment, sPAP decreased by 7.4 ± 14.3 mmHg, p < 0.05. After 12 months, sPAP was reduced by 9.8 ± 18.5 mmHg as compared to baseline (p < 0.05).

Patients were classified in WHO functional class II (n = 10; 32% of patients) and III (n = 22; 68% of patients). Overall WHO class was 2.7 ± 0.5, and tended to be lower after 3 and 12 months of treatment without reaching statistical significance (2.4 ± 0.5, and 2.6 ± 0.5 respectively) indicating improved exercise tolerance.

**Figure 1:**
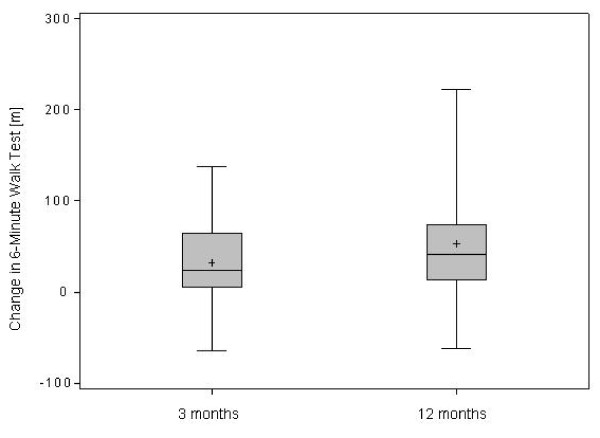
**Changes in Six-Minute Walk Test (6-MWT) after 3 and 12 months of treatment in 32 patients with IPAH. (p < 0.01 for both as compared to baseline)**.

Consistently in patients, 6-MWT was reduced between 110 to 405 m (Table 1). During 3 and 12 months of treatment, 6-MWT increased significantly (Figure 1).

**Table 1:** Pulmonary Physiologic Characteristics and Pulmonary Hypertension Characteristics in 32 patients with IPAH.

MEF_75_, % predicted	80.0 ± 20.4
MEF_50_, % predicted	65.0 ± 25.4
MEF_25_, % predicted	44.5 ± 23.6
VC, % predicted	90.2 ± 19.8
FEV_1_, % predicted	85.5 ± 15.8
R_tot_, kPa × s × L^-1^	0.3 ± 0.2
RV, % predicted	109.8 ± 21.4
TLC, % predicted	95.6 ± 12.5
sPAP, mmHg	79.4 ± 19.1
6-MWT, m	363.8 ± 125.2
NT-proBNP, ng/L	1427 ± 2162.7

6-MWT = six minute walk test; FEV_1 _= forced expiratory volume within first second; MEF75, 50, 25 = maximal expiratory flow at 75%, 50%, 25% of remaining VC; NT-proBNP = N-terminal-pro-B-type natriuretic peptide serum level; RV = residual volume, R_tot _= airway resistance; sPAP = systolic pulmonary artery pressure as determined from tricuspid regurgitation velocitiy during echocardiography, TLC = total lung capacity, VC = vital capacity. Data are presented as mean ± SD.

### Pulmonary function

In the present IPAH patients, lung volumes and airway resistance were within normal limits (Table 1). There was no significant change in vital capacity (VC), forced expiratory volume in 1 second (FEV_1_) and airway resistance (R_tot_) during 3 or 12 months respectively (data not shown).

**Figure 2:**
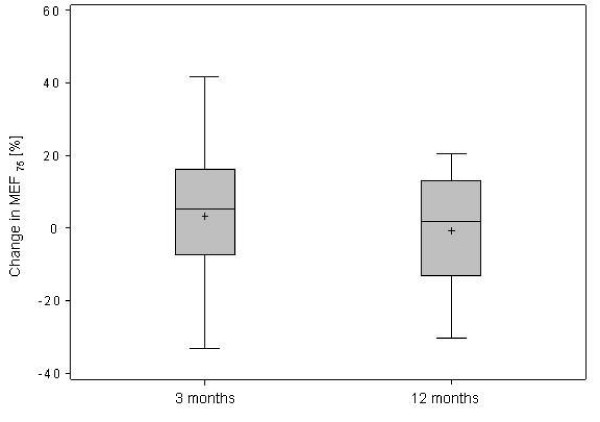
**Changes in maximal expiratory flow (MEF) at (a) 75%, (b) 50%, (c) 25% of remaining vital capacity after 3 and 12 months of treatment in 32 patients with IPAH**.

**Figure 3:**
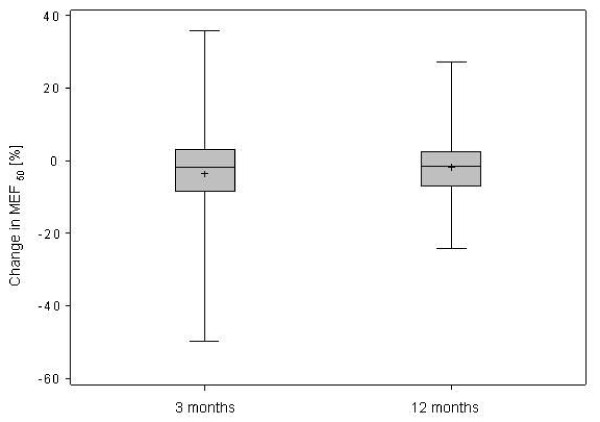
**Changes in maximal expiratory flow (MEF) at (a) 75%, (b) 50%, (c) 25% of remaining vital capacity after 3 and 12 months of treatment in 32 patients with IPAH**.

**Figure 4:**
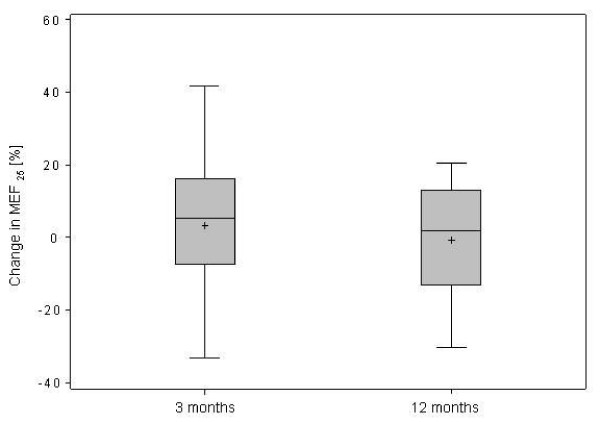
**Changes in maximal expiratory flow (MEF) at (a) 75%, (b) 50%, (c) 25% of remaining vital capacity after 3 and 12 months of treatment in 32 patients with IPAH**.

However, expiratory airflow during the second half of the expiratory phase was reduced, indicating peripheral airway obstruction (Table 1). After 3 and 12 months of treatment, the limitation in expiratory air flows persisted (Figure 2,3 and 4).

The residual volume (RV) and total lung capacity (TLC) at baseline (Table 1) remained without significant change during 3 and 12 months of treatment: RV (96.9 ± 13.8 and 95.3 ± 14.9% predicted) and in TLC (109.9 ± 24.2 and 113.4 ± 25.4% predicted) after 3 and 12 months, respectively.

### Serum biomarker

As compared to the increased NT-proBNP serum levels (Table 1) before treatment, NT-proBNP serum levels tended to be reduced by 122.4 ± 326.9 ng × L^-1 ^after 3 months treatment and by 529 ± 1942.2 ng × L^-1 ^after 12 months without reaching statistical significance (p = 0.11).

### Medication

In 2 patients, the oral PDE-5 inhibitor sildenafil was added to bosentan during the 12 months study period. On comparison between patients on bosentan monotherapy throughout the 12 months study period, and patients receiving additional sildenafil, the results in lung function testing did not differ. However, the statistical power of 2 observations is obviously poor.

## Discussion

The major findings of the present study in 32 IPAH patients are *(1) *the reduction in expiratory airflows similar to our previous observations [2]. This finding indicates peripheral airway obstruction. *(2) *The initiation of the ET-1 receptor antagonist bosentan increased exercise tolerance (6-MWT) and lowered systolic PAP and NT-proBNP serum levels during 3 and 12 months of treatment. *(3) *Independently from this significant improvement in disease severity, expiratory airflow limitation persisted.

### Peripheral airway obstruction in IPAH

In the present patients, expiratory airflows were decreased, particularly during the effort independent end expiratory portion of the flow-volume curve obtained at lower values of VC [16]. The expiratory airflow limitation together with a tendency towards increased RV in the present patients are in agreement with previous studies in PAH-patients [2,4,17,18]. However, the cause of expiratory airflow limitation in IPAH patients is unknown.

It might be speculated that the increased production of cytokines, growth mediators in the pulmonary vasculature in IPAH also contributes to proliferation in adjacent small airways. In a small study in 11 patients with IPAH showing airflow limitation at the lower part of VC was associated with histological airway narrowing, bronchial wall thickening, and lymphocyte infiltrates, thereby supporting earlier observations after necropsy [3,17].

On the other hand in 22 patients with IPAH, a single inhalation of beta-2-agonsists resulted in acute (however incomplete) reversal of airway obstruction [19]. This has first been described in children with pulmonary hypertension and Eisenmenger's syndrome [20]. Similarly in adults with IPAH, the inhalation of beta-2-agonist lead to an increase in FEV_1 _and MEF_50 _[19]. Interestingly in the latter study, the application of 2 puffs of 100 μg albuterol resulted in an acute increase in CO, stroke volume, mixed venous oxygen saturation, and arterial oxygen tension as well as a significant decrease in PVR, with the heart rate unchanged [20]. These findings, although derived from a small cohort, indicate that the long-term effects of inhaled beta-2-agonists in adults with IPAH might merit evaluation as an addition to the approved pharmacological interventions, especially endothelin receptor antagonists.

### Endothelin receptor antagonist treatment in IPAH patients

A major beneficial effect of bosentan therapy in the present patients is the improvement in exercise tolerance by 32 m during the first 3 months. This finding was similar to previous studies. In 21 patients with IPAH or scleroderma associated PAH and randomly assigned to bosentan, the 6-MWT increased by 70 m after 12 weeks as compared to baseline, whereas 6-MWT decreased by 6 m in 11 patients on placebo [9]. In another trial randomizing 213 patients in 27 study sites with IPAH or collagen-associated PAH to either bosentan or placebo, the 6-MWT improved by 36 m after 16 weeks of bosentan therapy as compared to a 6-MWT reduction of 8 m in the placebo group [10]. However, in a recent study in less compromised patients categorized in NYHA functional class II, the 93 patients receiving bosentan showed significant improvement in PVR, but not in 6-MWT, after 26 weeks of bosentan [21].

Extending the treatment period to 12 months in the present study, resulted in a further increase in 6-MWT by 53 m as compared to baseline. This finding is in accordance with the scarce data on long-term bosentan treatment [22]. In a retrospective analysis of a single center, 59 IPAH patients in NYHA functional class III/IV, 6-MWT improved significantly from 349 to 399 m at the end of 12 months bosentan treatment [22].

In parallel to the increased exercise tolerance, hemodynamics improved in the present patients. The sPAP was significantly reduced by 9% and 12% as compared to baseline after 3 and 12 months, respectively. Although the value of estimating sPAP from echocardiography has been debated as a marker of disease severity in IPAH [12], the present decrease in sPAP during 3 and 12 months supports the positive hemodynamic effects of the treatment with the vasodilator bosentan.

Moreover during 3 and 12 months of bosentan in the present IPAH-patients, the NT-proBNP serum levels, tended to be lowered by 8% and 37% from baseline without reaching statistical significance (p = 0.11). Previously, BNP serum levels have been shown to correlate with severity of disease and to be independent predictors of survival [23]. However, only limited and inconclusive data is available concerning the effects of long-term bosentan treatment on BNP serum levels [24]. Lately, after 16 weeks of bosentan in 12 PAH patients, BNP tended to be lower without reaching statistical significance [25].

Thus, in the present and in previous patients long-term treatment with bosentan improved severity of disease as assessed by exercise tolerance, hemodynamics and serum biomarker levels.

### Bronchial and parenchymal effects of endothelin receptor antagonist treatment

The ET-1 is a potent mediator of vasoconstriction and proliferation in the pulmonary vasculature [8]. ET-1 promotes pulmonary vascular and interstitial remodelling, causing smooth muscle proliferation, lung fibroblast activation, and proliferation of extracellular matrix deposition and contraction [26,27]. Moreover, ET-1 owns strong bronchoconstrictor properties, stimulates mucus secretion and mucosal edema, and may also exert pro-inflammatory effects [28].

Consequently, treatment with bosentan inhibits the eosinophilic reaction in the bronchial epithelium in an asthma model in rats [28]. Moreover, in rat tracheal allografts, bosentan ameliorates fibrous airway obstruction [29], and could reduces the progression of advanced airway disease if combined with the angiotensin-converting enzyme inhibitor ramipril [29].

Given the very limited data from animal studies, there is no lung function data of airway narrowing in neither animal nor clinical application of bosentan available.

This is the first study to address this question. The present findings show that expiratory airflow limitation persists during long-term ET-1 receptor antagonist treatment in patients with IPAH.

This is in concert with the recent observation in patients with significant COPD and consecutive pulmonary hypertension (i.e. Venice WHO group III), where airway obstruction was unchanged after 12 weeks of bosentan [30].

## Conclusion

This is the first study evaluating the effects of ET-I receptor antagonist therapy on lung function in patients with IPAH. Significant expiratory airflow limitation indicating peripheral airway obstruction was found. During 3 and 12 months of bosentan treatment, the markers of hemodynamic, functional and serum biomarker disease severity improved. However, expiratory airflow limitation persisted.

Given the still suboptimal therapeutical options to improve the functional state in IPAH patients, the underlying mechanisms and possible interventions of peripheral airway obstruction should be further evaluated.

## Competing interests

AF has participated in multi-centre studies sponsored by Actelion Pharmaceuticals, Freiburg, Germany. AF has received travel support and speakers fees from Actelion Pharmaceuticals.

ASD no competing interest.

DR has received travel support from Actelion Pharmaceuticals.

FJM has participated in multi-center studies sponsored by Actelion Pharmaceuticals. FJM has served in an advisory board for Actelion Pharmaceuticals, and he has received travel support and speakers fees from Actelion Pharmaceuticals.

HAK no conflict interest.

MMB has participated in multi-center studies sponsored by Actelion Pharmaceuticals. MMB has received travel support and speakers fees from Actelion Pharmaceuticals.

MV has received travel support and speakers fees from Actelion Pharmaceuticals.

TB no competing interest.

## Authors' contributions

AF interpreted the data and drafted the manuscript. ASD conceived and designed the study, acquired the data, interpreted the data and drafted the manuscript. DR acquired, interpreted the data, and drafted and revised the manuscript. FJM conceived, coordinated and designed the study, acquired and interpreted the data, and drafted and revised the manuscript. HAK interpreted the data and drafted the manuscript. MMB drafted the manuscript. MV interpreted the data and drafted the manuscript. TB participated in the design of the study, interpreted the data, and performed statistical analysis. All authors have read and approved the final manuscript.
